# Complete Genome Sequence of the Lytic Bacteriophage Phab24, Which Infects Clinical Strains of the Nosocomial Pathogen Acinetobacter baumannii

**DOI:** 10.1128/MRA.00669-21

**Published:** 2021-10-07

**Authors:** Belinda Loh, Xiaoqing Wang, Xiaoting Hua, Hou Wei Chook, Long Ma, Liwei Zhang, Prasanth Manohar, Yijie Jin, Sebastian Leptihn

**Affiliations:** a Independent Researcher, Munich, Germany; b Zhejiang University-University of Edinburgh (ZJU-UoE) Institute, Zhejiang University, International Campus, Haining, Zhejiang, People’s Republic of China; c Department of Infectious Diseases, Sir Run Run Shaw Hospital, Zhejiang University School of Medicine, Hangzhou, Zhejiang, People’s Republic of China; d College of Medicine & Veterinary Medicine, University of Edinburgh Medical School, Edinburgh, United Kingdom; Queens College CUNY

## Abstract

Phab24 was isolated from river water in Zhejiang Province, China, and exhibits lytic activity against clinical isolates of the nosocomial pathogen Acinetobacter baumannii (X. Wang, B. Loh, Y. Yu, X. Hua, et al., bioRxiv 2021.07.23.453473, 2021, https://doi.org/10.1101/2021.07.23.453473). The bacteriophage belongs to the *Myoviridae* family and has a double-stranded DNA (dsDNA) genome sequence that is 93,604 bp long, containing 172 open reading frames (ORFs).

## ANNOUNCEMENT

**FIG 1:**
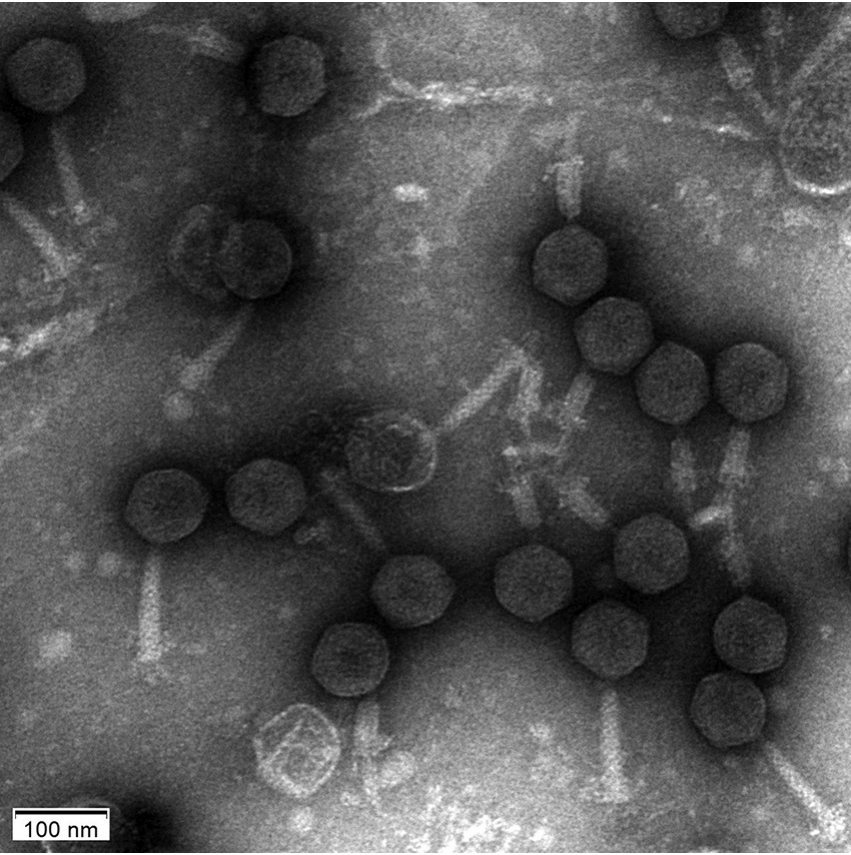
Transmission electron micrograph of Acinetobacter baumannii phage Phab24. Negative staining was conducted with 2% uranyl acetate. Bar = 100 nm.

Multidrug-resistant Acinetobacter baumannii is classified by the WHO as a top priority pathogen and is the causative agent of nosocomial infections (1). With rapidly increasing numbers of antibiotic-resistant bacteria, phage therapy has become a promising alternative approach to treat multidrug-resistant bacterial infections (2–4). Phab24 (phage Acinetobacter baumannii number 24) was isolated from river water in Haining, Zhejiang Province, China (120.605111°E, 30.481146°N). Water samples were collected according to G. Marpmann, using polypropylene tubes instead of glass containers (5). The samples were filtered through 0.45-μm membranes and enriched using a culture of the colistin-resistant strain A. baumannii XH198, following the protocol described in the Phage Discovery Guide (https://seaphagesphagediscoveryguide.helpdocsonline.com/home) (6). The phage was purified three times on double-agar overlay plates. Negative-staining transmission electron microscopy indicates that it belongs to the family *Myoviridae* in the order *Caudovirales* (Fig. 1).

Phages from a single plaque were replicated in the bacterial host grown in Mueller-Hinton broth with 10 mM MgSO_4_. After overnight incubation at 37°C, bacterial cells were removed by centrifugation, and the supernatant containing the phages was filtered through a 0.22-μm membrane. From the filtrate, phage DNA was extracted using the Biomed virus rapid DNA/RNA kit (Beijing, China). Libraries for sequencing were prepared using the NEBNext Ultra II DNA library prep kit for Illumina, and the genome was sequenced using the Illumina HiSeq platform. The read lengths of the raw data were 150 bp (total, 1,178,242,500 bp). Assembly was conducted using the genome assembly pipeline Unicycler v0.4.8 (7), which includes Pilon v1.2.3 for polishing, and SPAdes v3.13.0, which contains a SPAdes read error correction module for quality control (QC). The circularity of the genome sequence was also determined using Unicycler, which automatically identified and trimmed the overlapping ends. Genome annotation was conducted using the CPT Galaxy and Web Apollo interfaces (8). tRNAs were predicted using ARAGORN v2.36 (9) and tRNAscan-SE v2.0 (10). Open reading frame (ORF) prediction was performed using GLIMMER v3 (11) and MetaGeneAnnotator v1.0 (12), which were then manually validated by conducting BLAST v2.9.0 searches (13) (accessed on 18 June 2021) against the NCBI nonredundant and Swiss-Prot databases and the bacterial virulence factor database VFDB (accessed on 3 November 2020) (14, 15). Default parameters were used unless stated otherwise.

Phab24 has a double-stranded DNA (dsDNA) circular genome sequence of 93,604 bp with a GC content of 33.65%. The genome of Phab24 is predicted to encode 172 proteins, out of which 34 align with identified phage genes. BLAST analysis revealed Phab24 to be a novel phage, its closest relative being Acinetobacter phage BS46, with 64% sequence coverage (16). Several genes are dissimilar; in particular, the tail fiber protein of Phab24 has 56% sequence similarity to that of phage BS46, indicating that Phab24 might have a different host range compared to BS46. As no lysogeny-related genes were found in the Phab24 genome, together with the absence of toxins or virulence factors, Phab24 might be a suitable candidate for phage therapy.

### Data availability.

The complete genome sequence of Phab24 has been deposited into GenBank under the accession number MZ477002.1 and the SRA accession number SRS9702366.
